# Cysteine Glutathionylation Acts as a Redox Switch in Endothelial Cells

**DOI:** 10.3390/antiox8080315

**Published:** 2019-08-16

**Authors:** Agathe Lermant, Colin E. Murdoch

**Affiliations:** Systems Medicine, School of Medicine, University of Dundee, Dundee, Scotland DD1 9SY, UK

**Keywords:** S-glutathionylation, endothelial cells, cardiovascular diseases, glutathione, oxidative stress, reactive oxygen and nitrogen species, oxidative post-translational modifications, signal transduction, redox

## Abstract

Oxidative post-translational modifications (oxPTM) of receptors, enzymes, ion channels and transcription factors play an important role in cell signaling. oxPTMs are a key way in which oxidative stress can influence cell behavior during diverse pathological settings such as cardiovascular diseases (CVD), cancer, neurodegeneration and inflammatory response. In addition, changes in oxPTM are likely to be ways in which low level reactive oxygen and nitrogen species (RONS) may contribute to redox signaling, exerting changes in physiological responses including angiogenesis, cardiac remodeling and embryogenesis. Among oxPTM, S-glutathionylation of reactive cysteines emerges as an important regulator of vascular homeostasis by modulating endothelial cell (EC) responses to their local redox environment. This review summarizes the latest findings of S-glutathionylated proteins in major EC pathways, and the functional consequences on vascular pathophysiology. This review highlights the diversity of molecules affected by S-glutathionylation, and the complex consequences on EC function, thereby demonstrating an intricate dual role of RONS-induced S-glutathionylation in maintaining vascular homeostasis and participating in various pathological processes.

## 1. Introduction

Covering vascular lumen walls, endothelial cells (ECs) are a central component building the interface between blood and underlying tissue. ECs act on multiple processes to ensure the maintenance of systemic vascular homeostasis, including the regulation of blood pressure, haemostasis, tissue vascularization and inflammation. Endothelial dysfunction is thus involved in a wide range of pathologies, especially cardiovascular diseases (CVDs) such as diabetes, hypertension and peripheral arterial disease. Importantly, the direct contact of ECs with blood circulation makes them highly sensitive to local oxygen levels. The role of oxidative stress and high levels of reactive oxygen and nitrogen species (RONS), resulting from ischemia/reperfusion, is now widely recognized in the onset of CVDs [1,2]. RONS alter EC functions via inducing post-translational modifications (PTM) on major signalling proteins involved in the main physiological processes. Those modifications significantly modulate protein structure and activity, which highlights the potential importance of local redox environment for driving EC functions in both physiological and pathological processes. Among those modifications, S-glutathionylation emerges as an important modulator of EC behavior under oxidative stress.

The harmful effects of oxPTM have been widely related to pathological processes. However, attempts to develop antioxidant therapies to reduce oxidative damages and resolve CVDs have not met the success expected [3]. Moreover, some antioxidant molecules reversing oxPTM, and more precisely S-glutathionylation, recently appeared to impair key EC functions under oxidative stress such as post-ischemia revascularization [4]. More generally, antioxidants appear to contribute to pathological processes such as lung diseases [5,6]. Further evidence for antioxidant treatment leading to adverse effects comes from a randomized clinical trial for antioxidant use in pregnancy disorder, preeclampsia. This trial reported a surprising secondary outcome, in which antioxidant treatment (Vitamin C and E) caused lower birth weight with no protection against preeclampsia [7]. Taken together these findings reveal an essential underpinning role of basal RONS levels in the maintenance of vascular homeostasis. Our understanding of how S-glutathionylation affects EC function is developing. However, the precise implications of this modification in physiological processes are not fully understood, and often lead to contradictory findings. This complexity underlies a double-edge sword role of S-glutathionylation in response to oxidative stress and suggests an example of how an ox-PTM can contribute to both physiological and pathological processes by altering interlinked signaling pathways. It is then of critical importance to fully understand the mechanisms by which oxPTM like S-glutathionylation can influence EC functions to better appreciate the complex relation between oxidative stress and CVDs, and potentially rethink our therapeutic approaches.

In this context, this review will summarize the latest insights on the role of glutathionylated proteins in the regulation of major EC pathways, and their implications in the main physiological and pathological processes linked with vascular homeostasis.

## 2. Protein S-Glutathionylation

After translation, proteins undergo various modifications including palmitoylation, phosphorylation or hydroxylation, which alter protein function through changes in cellular location, activity or stability. Cysteinyl residues are especially sensitive to levels of oxygen and nitrogen species (RONS), resulting in a range of oxidative post-translational modifications (PTM) including S-nitrosylation, S-sulfenylation and S-glutathionylation.

**Figure 1:**
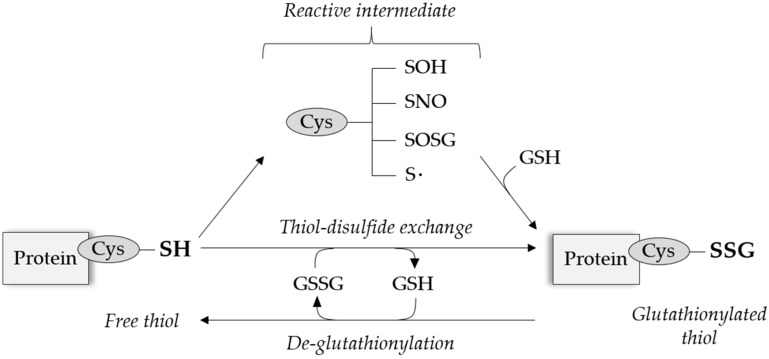
General mechanisms of reversible protein S-glutathionylation. Free thiols on reactive cysteinyl residues can be modified after the formation of an intermediate thiol derivative, or more rarely undergo direct thiol-disulfide exchange. S-glutathionylation can be reversed by the action of thiol-modifying enzymes, e.g., glutaredoxin (Grx). Cys = cysteine, GSH = glutathione.

Under physiological RONS levels, the antioxidant molecule glutathione (GSH) can bind to specific reactive cysteinyl residues via the creation of reversible disulfide bonds depending on the cysteine position and redox potential. This process of S-glutathionylation can be reversed by thiol modifying enzymes, predominantly glutaredoxin (Grx), and appears as a protective mechanism against permanent protein damage following irreversible cysteine oxidation, summarized in Figure 1.

The different molecular mechanisms involved in S-glutathionylation PTM have been extensively reviewed [8,9]. Beyond its protective functions, S-glutathionylation can directly modulate the structure and activity of redox sensitive proteins by modulating their global molecular mass and charge, or preventing other molecules to bind to their active sites [10]. Targeting a wide-range of proteins involved in key cellular processes acting from transcriptional, structural to post-translational levels, S-glutathionylation is now emerging as a key regulator of major signaling pathways in combination with other posttranslational regulatory mechanisms [11,12]. In this review, we will focus on the literature demonstrating S-glutathionylation involvement in endothelial signaling pathways and thus its importance in cardiovascular diseases. The wide range of proteins with redox sensitive thiols involved in EC function, and the impact of S-glutathionylation on the corresponding protein activity are summarized in Table 1. This gives an overview of the breadth and impact that S-glutathionylation can have on EC function.

## 3. Role of Endothelium in Vascular Physiology

**Figure 2:**
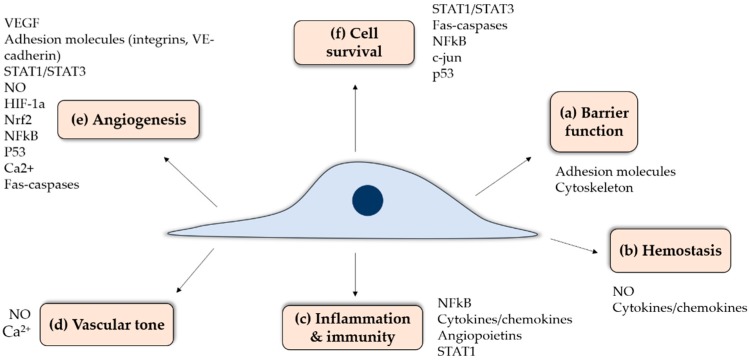
Schematic representation of the main endothelial cell functions and associated molecular pathways which are suspectable to S-glutathionylation.

Endothelial cells (ECs) are a specialized tissue lining the lumen of all blood vessels. At the interface between circulating blood and underlying tissues, ECs have multiple functions (Figure 2) including: (a) maintenance of a selective permeability barrier between both compartments to coordinate the passage of macromolecules, ions or signaling molecules through endothelial junctions or intracellular clefts; (b) maintenance of hemostasis through tight spatial and temporal interplays between pro-thrombotic, anti-coagulant, anti-platelet and fibrinolytic activities and the regulation of blood cell-vessel wall interactions; (c) involvement in innate and adaptive immune responses and inflammation; (d) modulation of vascular smooth muscle tone and thus blood flow distribution and control of blood pressure; (e) formation of new blood vessels, or angiogenesis, through the regulation of vessel wall cell proliferation and apoptosis; and (f) a contribution to the maintenance of a quiescent, differentiated vascular smooth muscle phenotype. This broad array of EC functions is ensured by multiple actors involved in orchestrated signalling pathways to maintain vascular homeostasis (Figure 2). Figure 2 depicts some of the key proteins involved in EC function, which are modification by S-glutathionylation that will be reviewed herein. Since ECs are found in all organs, a proper interplay between these pathways is essential to a wide range of physiological functions. In contrast, EC activation or dysfunction can lead to various pathologies. Endothelial activation is a term used to describe reversible phenotypical changes of the endothelium, notably an increase in adhesion molecule expression, and includes a wide spectrum of events such as variations in signaling cascades, transcription factors, gene expression and the cytoskeleton. Endothelial dysfunction entails pathophysiological dysregulation, which is at the extreme range of endothelial activation where it becomes permanent or maladaptive, contributing to the development of pathological conditions. Endothelial dysfunction is often present many years before the manifestation of clinical disease symptoms, for example in the case of type-2 diabetes and Alzheimer’s disease.

## 4. Transcription Regulation by S-Glutathionylation: Epigenetics Regulators and Transcription Factors

### 4.1. Epigenetic Regulators

**Figure 3:**
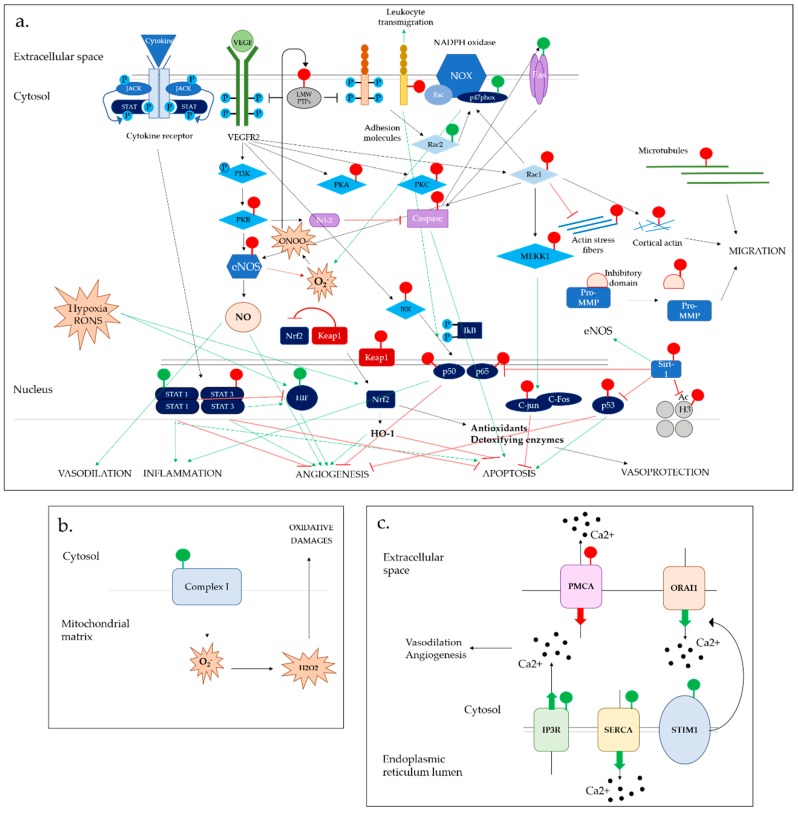
Overview of the interlinked effects of S-glutathionylation in major EC functions via the alteration of (**a**) major signaling pathways in cytosol and nucleus (**b**) RONS formation in mitochondria (**c**) calcium-dependent signaling in the endoplasmic reticulum.

The importance of epigenetics in EC function and related complex diseases is meeting a growing interest, highlighted by the recent emergence of whole genome studies. The modification of histone methylation state plays a key role in EC function by acting upstream of gene transcription, and its implications in cardiovascular pathologies [76]. Importantly, epigenetics appears as an essential player in EC progenitor function, therefore regulating vascular repair processes [77,78]. Histone modifications are particularly important for regulating the expression of key EC genes as the endothelial nitric oxide synthase (eNOS) and the vascular endothelial growth factor 1 (VEGFR1), therefore playing a pivotal role in vascular homeostasis and angiogenesis (Figure 3a) [79,80,81]. Oxidative stress is known to alter chromatin structure, and S-glutathionylation occurs in histones and histone-modifying enzymes [82]. However, how S-glutathionylation directly relates to epigenetic-induced onset of cardiovascular diseases (CVD) warrants further assessment since the following factors undergo oxPTM.

#### 4.1.1. Histone Proteins

In eukaryotes, the chromatin is packaged in individual units called nucleosomes. Histones, the building bricks of nucleosomes, are subject to various PTM which directly impact the chromatin structure and, as a result, gene expression [83]. Although cysteine residues have been identified in histone H3 [84], studies on redox regulation processes such as S-glutathionylation on histones have been halted by challenging technical requirements. Only recently has S-glutathionylation been identified on histone H3 in cancerous cells [13]. S-glutathionylation of histone H3 has been confirmed and further characterized by Olaso et al., who identified a target cysteine residue on the protein [14]. Despite altering the nucleosome structure and, therefore, chromatin compaction, the exact functional implications of S-glutathionylation on chromatin structure are not widely appreciated. While Garcia et al. suggested that histone H3 S-glutathionylation opens chromatin structure and promotes gene expression [14], another study claimed opposite effects [85]. These discrepancies may be due to the difficulty in studying highly reversible oxidative modifications. Further studies are required to elucidate the functional consequences of histone H3 S-glutathionylation on gene expression, and the potential existence of other S-glutathionylation modifications on other reactive Cys in histone H3 previously identified [14,84].

#### 4.1.2. Histone-Modifying Enzymes

The effect of S-glutathionylation on the modulation of chromatin structure can be appreciated indirectly, via the modification of histone-modifying enzymes. Particularly, RONS are known to decrease histone deacetylase (HDAC) activity, notably via nitrosylation, favoring gene transcription [86,87]. However, the role of S-glutathionylation in the regulation of histone acetylation state and implications in EC physiology is only starting to be elucidated with sirtuins. This family of proteins plays an important role in the regulation of endothelial cell homeostasis via deacetylation of multiple targets [88]. In addition to its role in angiogenesis and diastolic functions [89,90], sirtuin-1 (SIRT1), also called “longevity protein”, appears to play a protective role in EC response to oxidative stress by reducing RONS levels. While p65 gene deacetylation by SIRT1 inhibits nuclear factor kappa-light-chain-enhancer of activated B cells (NFκB) signaling [91,92], the same process enhances eNOS activity, therefore promoting nitric oxide (NO) production and vasodilation while reducing RONS production [93,94]. Three cysteines have been identified as susceptible to thiol modifications on SIRT1 [15], and the presence of S-glutathionylation has been confirmed [16]. SIRT1 S-glutathionylation appears to inhibit enzymatic activity by altering protein structure and binding to nicotinamide adenine dinucleotide (NAD+), leading to cell senescence and apoptosis (Figure 3a) [17].

### 4.2. Transcription Factors

S-glutathionylation regulates various transcription factors in ECs, most of the time by inhibiting their activity and, therefore, silencing the expression of downstream targets (Figure 3a). Interestingly, some exceptions were reported where this modification had opposite effects, enhancing gene expression. The redox regulation of transcription factors illustrates the complexity of S-glutathionylation acting to switch effects in multiple signaling pathways and its potential clinical implications.

#### 4.2.1. S-Glutathionylation-Mediated Inhibition of Transcription Factors

NFκB transcription factor complex is mostly known for its pro-inflammatory properties, characterized in ECs by a promotion of vascular permeability and leukocyte recruitment [95]. Besides promoting inflammation, NFκB also plays a key role in the regulation of angiogenesis and cell survival, notably via Wnt5a signaling pathway [4,96].

S-glutathionylation regulates various components of the NFκB pathway. NFκB major subunits p50 and p65 activity are inhibited by S-glutathionylation, preventing DNA-binding or nuclear translocation and downstream gene transcription [18,19]. S-glutathionylation was also reported in the protein kinase IKKb, which impaired its kinase activity and, therefore, further promotes the p65/p50 sequestration in the cytosol [31]. The consequences of S-glutathionylation-induced inhibition of NFκB have been demonstrated in various inflammatory diseases and have been extended to ECs. Recent studies highlighted an essential role of this process for neovascularization response. This is in accordance with the recognized antiangiogenic properties of NFκB signaling via the production of the antiangiogenic factor soluble Vascular growth factor receptor 1 (sVEGFR1) (Figure 3a). [42,43]. Previous studies, however, suggested that NFκB inhibition by S-glutathionylation in mice fibroblasts promoted cell hypoxic apoptosis [44].

As a component of the AP-1 transcription factor complex, c-Jun protein promotes cell proliferation and angiogenesis in ECs. However, its precise roles in cell survival are still controversial. The signaling cascade of c-Jun is known to be susceptible to redox regulation [43,44,45,46,47,48,49,50,51,52,53,54,55,56,57,58,59,60,61,62,63,64,65,66,67,68,69,70,71,72,73,74,75,76,77,78,79,80,81,82,83,84,85,86,87,88,89,90,91,92,93,94,95,96,97,98,99,100]. Displaying a particularly high redox potential, S-glutathionylation of its Cys269 residue has been extensively reported (Figure 3a) [20,21]. This oxPTM inhibits the DNA binding of c-jun, with the functional consequence in ECs and may contribute to invasive breast cancer and/or pulmonary hypertension [43,100].

p53 is gatekeeper of a wide range of cellular pathways, mostly known for its antitumorigenic properties preventing abnormal cells proliferating. However, the role of p53 in non-cancerous cells such as ECs has recently attracted interest due to its crucial implications in angiogenesis, apoptosis and vascular dysfunction [101]. p53 S-glutathionylation inhibition of DNA binding and protein dimerization has been reported in cancerous cells [22], having potential implications in EC function and vascular pathologies associated with defects in angiogenesis regulation.

Nuclear factor (erythroid-derived 2)-like 2 (Nrf2), a basic redox-regulated leucine zipper protein, is another major transcriptional factor that plays fundamental roles in EC functions including essential anti-angiogenic activity. In response to oxidative stress, Nrf2 mediates antioxidant and anti-inflammatory signals providing crucial cytoprotective effects in many cells types including the endothelium(Figure 3a) [102]. The range of cytoprotective proteins induced by Nrf2 activation include NADPH quinone oxidoreductase 1, sulfiredoxin 1, Heme oxygenase-1 and glutathione S-transferases. In addition, Nrf2 regulates angiogenesis post ischemia [103]. A novel role of S-glutathionylation in modulating Nrf2 activity was recently reported in various cell types, where S-glutathionylation of its inhibitor Keap1 results in Nrf2 nuclear translocation and expression of its downstream targets [29,30]. Those findings highlight the importance of S-glutathionylation in cell response to oxidative stress.

#### 4.2.2. S-Glutathionylation-Mediated Activation of Transcription Factors

Hypoxia-inducible factor 1-alpha (HIF-1α) is the major regulator of oxygen homeostasis in ECs, and promotes angiogenic signaling in response to hypoxia [104]. Alterations in the protein activity, therefore, play a crucial role in severe pathological states associated with ischemia [105]. The role of HIF-1a S-glutathionylation was extensively studied in cancerous cells. In the hypoxic tumor environment, this modification stabilizes the protein and, therefore, promotes tumor growth and angiogenesis (Figure 3a) [23]. Accordingly, the promotion of S-glutathionylation of HIF-1α in endothelial cells was found to accelerate ischemic revascularization via VEGF-A production in animal models [24], showing important implications in the mechanism of recovery from ischemic injury.

#### 4.2.3. Opposite Effects of S-Glutathionylation on Various Signal Transducer and Activator of Transcription (STAT) Proteins

The JAK/STAT signaling pathway is activated in response to cytokines and growth factors and stimulates a wide range of physiological processes including inflammation, apoptosis, differentiation, cell migration and proliferation. In this pathway, Signal Transducer and Activator of Transcription (STAT) family proteins are intracellular transcription factors phosphorylated by JACK, and translocated to the nucleus to promote gene expression (Figure 3a) [106]. These include STAT1 and STAT3, activated not only by cytokine receptors but also by Vascular Endothelial Growth Factor (VEGF) signaling in endothelial cells. STAT pathway, therefore, plays a role in angiogenic response and cell growth [107,108]. Beyond their role in angiogenesis, STATs are highly important in promoting EC inflammatory response, and are known to play a role in atherosclerosis progression [109,110].

S-glutathionylation of STAT3 prevents phosphorylation inhibiting DNA-binding capacities [25], and a more recent study identified two reactive cysteine residues subject to S-glutathionylation in STAT3 structure [26]. Carbon monoxide (CO)-mediated S-glutathionylation of the reactive cysteines under mild oxidative conditions exerts a cytoprotective mechanism, modulating pro-inflammatory signals in ECs [27]. S-glutathionylation of STAT1 was observed in microglial cells. Interestingly, the effects of this modification appear contrary to the ones observed with STAT3, as STAT1 phosphorylation was not impaired by S-gluathionylation and activity was enhanced [26]. STAT3 and STAT1 have been shown to have antagonistic effects on angiogenesis and cell proliferation in endothelial cells [111,112]. Although inducing opposite effects on the two STAT proteins, S-glutathionylation seems to exhibit a general inhibitory role on JACK-STAT-mediated angiogenesis.

## 5. Redox Control of Phosphorylation by S-Glutathionylation: Phosphatases, GTPases and Kinases

Protein phosphorylation is a pivotal post-translational modification regulating cell signaling pathways. Phosphatase and kinase enzymes regulate the phosphorylation state of major proteins in EC signaling as receptors and downstream targets (Figure 3a). While cysteine oxidation is generally considered as an inhibitor of phosphatase activity, the effects on kinases requires further investigation [113,114]. The example of S-glutathionylation seems to follow this pattern and could bring novel insights into the redox regulation of phosphorylation in key EC signaling pathways and effects of oxidative stress in pathophysiological conditions.

### 5.1. Phosphatases

Protein tyrosine phosphatases (PTP) down-regulate key receptors by dephosphorylating kinase domains.

Low-molecular-weight protein tyrosine phosphatase (LMW-PTP) family proteins inhibit growth factors regulating cell growth and angiogenesis, but also inhibit focal adhesion kinase regulating cell migration. LMW-PTP, characterized by the presence of two reactive cysteines located in their catalytic domain, are widely recognized as a redox-dependent “molecular switche” in many cell types [115,116,117]. More specifically, S-glutathionylation was shown to inhibit LMW-PTP phosphatase activity in ECs and, therefore, appears essential for promoting angiogenesis and EC migration [32]. This same study demonstrated that this modification was mediated by VEGF-dependent ONOO^-^, highlighting its role in a negative feedback loop central to VEGF signaling.

PTP1B is a major regulator of EC proliferation through its binding and inactivation of the VEGFR2 receptor. Its phosphatase activity on tyrosine domains also applies to the VE-cadherin receptor, stabilizing cell–cell adhesion and, therefore, limiting vascular permeability [118]. S-glutathionylation inhibits PTP1B activity [33,34]. Although the functional consequences of this modification were not studied in directly in ECs, mice lacking PTP1B display enhanced angiogenic and revascularization capacities as well as an increased cardiac perfusion following myocardial infarction [119]. Further studies are needed to investigate the potential implications of PTP1B inhibition by S-glutathionylation in cardiovascular diseases related to VEGF and VE-cadherin signaling. S-glutathionylation could also appear as a key redox switch for the regulation of other PTPs such as HCPTPA, also involved in VEGF receptor inhibition [120].

### 5.2. GTPases

GTPases, hydrolyze GTP to provide the energy required for central physiological processes, play an important role in the regulation of vascular permeability and RONS production. Defects in vascular barrier function are a hallmark of vascular dysfunction, promoting leukocyte transmigration and chronic inflammation, and have been associated with various severe pathologies such as diabetes [121,122] or pulmonary disorders [123]. Enhanced RONS and S-glutathionylation levels appear to promote vascular permeability, suggesting redox control [35,123]. However, which components are S-glutathionylated and how this process affects vascular permeability is yet to be described in detail.

Small Rho GTPases act in a complex interplay to modulate the dynamics of the actin cytoskeleton, a central feature for EC barrier function and migration [124,125]. GTPases are recognized as redox-sensitive and generally inhibited by oxidative stress [38]. Among them, Ras-related C3 botulinum toxin substrate 1 (Rac1) S-glutathionylation was recently shown to alter the enzyme function, resulting in loss of cortical actin structure, increased stress fibers and cell–cell adherens junction disassembly [35]. On the contrary, another study evidenced the opposite effect on Rac2, on which S-glutathionylation appeared to enhance GTP-binding activity [37]. The Ras subfamily of GTPases S-glutathionylation was observed in ECs and smooth muscle cells (SMC). However, the biological effects on nucleotide exchange and protein activity remain controversial [87,89,90].

Beyond the regulation of vascular barrier integrity, further understanding of Rho GTPase redox regulation could provide important insights on EC response to oxidative stress through NADPH oxidase and eNOS activation [36,37,38,39,40,41,42,43,44,45,46,47,48,49,50,51,52,53,54,55,56,57,58,59,60,61,62,63,64,65,66,67,68,69,70,71,72,73,74,75,76,77,78,79,80,81,82,83,84,85,86,87,88,89,90,91,92,93,94,95,96,97,98,99,100,101,102,103,104,105,106,107,108,109,110,111,112,113,114,115,116,117,118,119,120,121,122,123,124,125,126] and, therefore, angiogenesis and cell migration [127].

### 5.3. Kinases

Kinase proteins are now widely recognized as redox-regulated, and more specifically subject to inhibition by S-glutathionylation due to the presence of a reactive cysteine residue in their catalytic domain [40]. It is notably the case for protein kinase B (PKB), which activity was maintained by Grx in various studies [42,43]. PKA, PKC and mitogen-activated protein kinase (MEK) are other examples of S-glutathionylation-induced inhibition of kinase activity [45,46,47,48,49,50,51,52,53,54,55,56,57,58,59,60,61,62,63,64,65,66,67,68,69,70,71,72,73,74,75,76,77,78,79,80,81,82,83,84,85,86,87,88,89,90,91,92,93,94,95,96,97,98].

Although precise biological implications of protein kinase redox regulation have not been extensively explored in ECs, the results showing that their enzymatic activities are importantly altered by S-glutathionylation could have crucial impacts for EC physiology. Indeed, the role of various kinases in key EC functions, especially the regulation of vascular barrier function and blood pressure, is well established [128,129].

## 6. S-Glutathionylation Effects on RONS Production

### 6.1. NADPH Oxidase Complex

In ECs, the major source of RONS is a NADPH oxidase (NOX) homologue. Upon activation by VEGF, ET-1, Angiotensin II (AngII) or Transforming Growth Factor β (TGFβ), the production of superoxide (O_2_^−^) and hydrogen peroxide from NOX regulates multiple redox-dependent pathways, especially those involved in the regulation of vascular tone and angiogenesis through NO inhibition [130,131,132] (Figure 3a). As a result, NOX-dependent RONS expression induces endothelial dysfunction and various cardiovascular pathologies such as hypertension, diabetes and cardiac failure [4,106,107,108,109,110,111,112,113,114,115,116,117,118,119,120,121,122,123,124,125,126,127,128,129,130,131,132,133,134,135]. The activation of the catalytic transmembrane unit relies on interactions between several cytosolic proteins, including cytosolic phox subunits and Rac1 [136,137]. In addition to Rac1 S-glutathionylation mentioned previously, p47phox S-glutathionylation was reported in neutrophils on three cysteines, in which the modification appears essential to drive sustained O_2_^−^ generation [46]. Interestingly, S-glutathionylation did not alter the protein phosphorylation state of p47phox, essential for its activation. As p47phox was identified as a key factor for RONS production in ECs stimulated by TNF-α [138], p47phox S-glutathionylation could then appear as an upstream redox switch promoting endothelial dysfunction through multiple target oxidation. However, further studies are needed to confirm that a similar effect is observed in EC despite some variations in vascular NOX properties compared to phagocytic cells [139]. A similar effect was observed on mitochondrial Complex I, another major source of O_2_^−^ in EC (Figure 3b) [47,140].

### 6.2. Endothelial Nitric Oxide Synthase System

eNOS is the major source of NO in ECs. This soluble gasotransmitter plays a central role in vascular homeostasis by regulating vascular tone, angiogenesis and maintenance of barrier integrity. The effects of RONS on eNOS activity are now generally recognized. Under oxidative stress, eNOS adopts a NADPH oxidase function and switches from NO to O_2_^−^ generation, which is a hallmark of multiple cardiovascular pathologies including atherosclerosis [141]. The role of eNOS S-glutathionylation in this process has recently met a growing interest. eNOS S-glutathionylation at the reductase site alters electron transfer and, therefore, uncouples eNOS, promoting O_2_^−^ over NO generation [48]. Since then, the role of S-glutathionylation has been further assessed [142] and shown in different pathologies such as hypoxia/reoxygenation injury [49] or necrotizing enterocolitis [50]. Importantly, eNOS S-glutathionylation is NADPH oxidase dependent [51,52], potentially contributing to NO inhibition resulting from O_2_^−^ production by NOS (Figure 3a).

## 7. S-Glutathionylation Effects on Ca^2+^ Homeostasis

Major functions of EC including angiogenesis, cell migration and growth, all depend on cytosolic calcium ion (Ca^2+^) concentrations ([Ca]i), tightly regulated by calcium-dependent channels located at the surface of the endoplasmic reticulum (ER), mitochondria and plasma membrane (Figure 3c) [143]. The effects of oxidative stress, and more precisely oxidized glutathione, on [Ca^2+^]_i_ modulation have been reported [53,144], suggesting S-glutathionylation as a potential regulator of cellular Ca^2+^ handling.

### 7.1. Calcium-Dependent IP3R & PMCA Channels

Lock et al. were the first to show that direct modification of inositol trisphosphate receptor (IP3R) and plasma membrane Ca^2+^ ATPase (PMCA) by S-glutathionylation have an effect on Ca^2+^ oscillatory patterns and [Ca^2+^]_i_ [56]. Interestingly, opposite effects were observed on each channel: while S-glutathionylation appeared to inhibit PMCA activity, it was shown to enhance IP3R activity, both increasing Ca^2+^ entry into aortic EC cytosol [56]. This latter effect was further explored in a later study which suggested that S-glutathionylation enhanced the receptor sensitivity to cytosolic Ca^2+^, leading to increased Ca^2+^ leaking into the cytosol [54]. Despite the identification of several cysteines potentially subject to S-glutathionylation in it the receptor structure, [55], whether this enhanced activity is due to direct protein modification or S-glutathionylation of accessory components is not known.

### 7.2. SERCA2b Calcium Pump

The sarco/endoplasmic reticulum calcium ATPase2 (SERCA2) pump promotes Ca^2+^ uptake in ER stores in ECs and SMC, directly regulating downstream processes involved in the maintenance of vascular homeostasis [145]. The importance of SERCA2 S-glutathionylation was first identified in SMC, in which NO-induced S-glutathionylation on one reactive cysteine enhanced protein uptake activity and muscle relaxation. S-glutathionylation of SERCA2 was well characterized, yet it is not clear how it leads to increased uptake by the pump [146].

This finding brought novel insights on the redox regulation of EC functions in response to hypoxia. Recent studies pointed out the essential role of SERCA S-glutathionylation in VEGF-induced EC migration via NOX signaling [57,147]. Thompson et al. further confirmed the physiological implications by showing that reversible SERCA2 S-glutathionylation was required for hypoxia-induced angiogenic responses in mouse models [58]. Another study proposed a multiplayer model where SERCA2 S-glutathionylation in EC and macrophages is essential for their interplay leading to angiogenic response [59]. Altogether, those results provide novel insights in the importance of redox-regulated Ca^2+^ store maintenance in VEGF-induced angiogenic response in EC.

The multiple effects of SERCA2 S-glutathionylation in SMC, EC and macrophages emphasize its importance in CVDs. This reversible modification appears as a protective mechanism against permanent SERCA oxidation, linked to severe conditions including atherosclerosis, cardiac dysfunctions, diabetes and impaired ischemic revascularization [90,132,134,135,136].

### 7.3. STIM1 Molecule and ORAI1 Channel

Stromal interaction molecule 1 (STIM1) senses Ca^2+^ contents of ER stores and replenishes them by activating calcium release-activated calcium channel protein 1 (Orai1) promoting Ca^2+^ entry into the cell [148]. S-glutathionylation was shown on one highly conserved reactive cysteine of STIM1, leading to protein oligomerization in fibroblasts. This process triggered a constitutive activation of Orai1, therefore, increasing global Ca^2+^ entry into intracellular stores. This sustained mitochondrial Ca^2+^ overload alters its function, resulting in cell death [63]. Highly expressed in EC, STIM1 is key to the maintenance of cell apoptosis and barrier integrity via both Ca^2+^ dependent and independent pathways and is, therefore, an important player in inflammation [130,132,133].

## 8. S-Glutathionylation Effects on Cell Death and Autophagy

### 8.1. Apoptotic Signalling

EC apoptosis is a central process of vascular homeostasis, as it maintains vasculature turnover and tightly regulates network formation during angiogenesis [149]. In addition to causing defects in vessel network formation, the dysregulation of apoptosis contributes to pathological conditions such as atherosclerosis via factors released by extracellular vesicles throughout the process [150]. Oxidative stress is regarded as a general mediator of EC apoptosis [151,152].

One aspect of the apoptotic process in EC involves the activation of the death receptor Fas [153] also involved in the maintenance of vascular integrity [154,155]. S-glutathionylation is suggested to upregulate the receptor activity and subsequent apoptotic signaling (Figure 3a) [64].

The apoptotic response is mediated by a cascade activation of the proteolytic enzymes cysteine-aspartic proteases (Caspases) downstream death receptors [153]. Caspase active sites, composed of cysteines, by definition are interesting targets for deciphering the role of redox-dependent thiol modifications such as S-glutathionylation in EC death receptor signaling pathway. The importance of thiol modifications in Caspases were first highlighted when a disulfide bond formation on Caspase-1 thiol inhibited its activity [156]. Pan et al. demonstrated that S-glutathionylation of Caspase-3 stabilizes the enzyme, inhibiting its cleavage required for activation, therefore, inhibiting apoptosis in EC [65]. Caspase-3 S-glutathionylation was further characterized in various cell types, in a study revealing two cysteines undergoing S-glutathionylation. While one is located in the enzymatic site, it remains to be discovered how the second one affects enzymatic activity [66]. Similarly, S-glutathionylation of caspase-8 in mice models seems to inhibit apoptosis, suggesting a protective mechanism against pathologies related to cell toxicity [67].

### 8.2. Autophagy

Growing evidence points out a protective role of EC autophagy in various physiological processes following oxidative stress and RONS production [157,158], as in ischemia/reperfusion injury [159], notably by promoting NO production [160]. Apoptosis and autophagy pathways are interlinked, and S-glutathionylation is likely to promote EC autophagy by upregulating the activity of autophagy-related protein Beclin-1 [68]. The altered function of Beclin-1, a major mediator of this process, was found to participate in various pathological processes such as atherosclerosis or hypertension promoting EC dysfunctions [161,162], pointing out the potential implications of S-glutathionylation in CVDs through autophagy regulation.

## 9. Redox Regulation of Cell Structure and Dynamics by S-Glutathionylation

### 9.1. Metalloproteases

Metalloprotease (MMP) enzymes are implicitly involved in angiogenesis by degrading endothelial cell matrix (ECM) components, permitting the endothelial cells to migrate within tissues. MMPs also contribute to angiogenesis releasing proangiogenic stimuli to further promote EC migration (Figure 3a) [163]. In addition to cancer, the dysregulation of MMP activity contributes to inflammation, and multiple cardiovascular conditions especially cardiac hypertrophy and atherosclerosis [164,165]. While previous studies pointed out the mechanisms of MMP redox regulation through the modulation of upstream mitogen-activated protein kinase (MAPK) pathway [166,167], others identified direct redox-dependent modifications of the MMP system. MMP activity is tightly regulated and activated in situ by cleavage of a pro-MMP form by tissue inhibitors of metalloproteinases (TIMPs). Promising results showed that S-glutathionylation of pro-MMP inhibitory domain could trigger the passage from latency to activated state, therefore, enhancing angiogenesis and vascular permeability (Figure 3a) in animal models [69,168].

### 9.2. Adhesion Proteins

Integrins are the major points of contact between ECs and their extracellular microenvironment, driving cell migration, proliferation and regulating vessel permeability. In addition to promoting cell adhesion and migration within the ECM, integrins act as receptors to trigger specific proangiogenic signaling pathways (Figure 3a) [169]. In leukocytes, thiol-based redox regulation of integrins modulate their structure and activity [170]. More precisely, S-glutathionylation of integrin α4 enhances the binding of neutrophils to ECs [171,172]. Despite Integrins wide range of biological functions, the physiological effects of EC integrins S-glutathionylation remain elusive. However, this is likely to contribute to endothelial activation, therefore, modulating the inflammatory response.

Vascular cell adhesion protein 1 and Intercellular Adhesion Molecule 1 (VCAM-1 and ICAM-1) are mainly expressed in ECs in response to pro-inflammatory cytokines and RONS. Adhesion proteins play a pivotal role in EC activation and binding to leukocytes, mediating leukocyte transmigration during inflammation and are, therefore, involved in inflammatory diseases such as rheumatoid arthritis [173,174]. Growing evidence also suggests a role of VCAM-1 in angiogenesis via VEGF activation, with subsequent implications in cancer progression [175]. A recent study suggested that Tumor necrosis factor α (TNF-α) induced ICAM-1 S-glutathionylation, altering protein folding and promoting its degradation [70]. The multiple cystine residues present on ICAM-1 make it particularly subject to oxidation processes, and a better understanding of redox effects on protein function could provide novel insights on the mechanisms involved in inflammatory diseases and cancer.

### 9.3. Cytoskeletal Proteins

Cytoskeletal dynamics, a key feature driving vascular angiogenesis and barrier function, are governed by polymerization and depolymerization of the two main component units, actin and tubulin. Both proteins are highly redox-sensitive and S-glutathionylation is now recognized to have a major impact on the regulation of polymerization-depolymerization cycles in various cell types [71,136,176]. Polymerization-depolymerization cycles have been better characterized in actin, on which S-glutathionylation modification on one reactive cysteine residue inhibits polymerization and, therefore, plays a key role in the regulation of cytoskeletal dynamics driving cell motility (Figure 3a) [71,72,73]. Similarly, glutathionylated microtubules appear depolymerized, promoting cell growth arrest [74,75]. Phenotypical effects were not investigated in ECs specifically. However, S-glutathionylation-induced changes in cytoskeletal reorganization could emerge as an important feature altering cell motility under oxidative stress.

## 10. Conclusions

S-glutathionylation has emerged as a novel redox switch altering the functions of EC through the modulation of key signaling pathways. Its importance and complexity in EC homeostasis and cardiovascular physiopathology is highlighted by recent insights on how S-glutathionylated protein functions are altered in interlinked molecular pathways at multiple levels (Figure 3).

It is now apparent that S-glutathionylation can coordinate gene transcription by modulating epigenetics and transcription factors. Studies investigating the impact of S-glutathionylation in epigenetics are still at their early stage, and further work is required to fully understand how it alters gene expression. However, current insights demonstrate that both histone proteins and histone-modifying enzymes are prone to S-glutathionylation, altering chromatin structure and gene expression. Given the number of various genes regulated by those processes, novel insights on the links between oxidative stress and epigenetics would allow a better understanding of EC signaling in response to RONS, and highlight novel factors involved in complex diseases for which genetic factors alone are not sufficient to explain. The effect of S-glutathionylation on transcription factors suggest differential effects on their activity depending on the protein targeted. Accordingly, it is difficult to define a general effect of this modification on EC physiology. However, the redox regulation of VEGF signaling appears central to the activation of downstream transcription pathways and the alteration of resulting EC functions.

S-glutathionylation exhibits a general inhibitory effect on enzymes by altering the structure of their catalytic site and impairing their activity. This has important repercussions when applied to phosphatases, GTPases and kinases, which are key signal transducers in ECs. The multiplicity of pathways regulated by enzymatic activity underlines the crucial importance of understanding the redox mechanisms dictating their behavior to better understand EC responses to oxidative stress.

As upstream mediators of S-glutathionylation, RONS themselves are subject to redox regulation. The two main RONS sources in EC, NOX and eNOS, are under tight redox control. The different effects of S-glutathionylation on RONS-producing systems have a general tendency to promote RONS production. This suggests a retroactive system, where RONS sustain their own production, which could importantly participate in oxidative stress-mediated EC dysfunction.

In respect to calcium signaling, S-glutathionylation tends to favor ion entry into the cell cytosol through reversible alteration of various player functions, except for the SERCA channel. Those results confirm that this modification plays a central role in Ca^2+^ homeostasis in EC, and that a tight regulation of RONS levels is required to ensure physiological Ca^2+^ levels in cellular stores allowing proper EC functions.

The opposing effects of S-glutathionylation on different apoptotic factors may explain the complex and diverse effect of oxidative stress on EC apoptosis.

The role of S-glutathionylation in EC autophagy is less studied, and we expect future work will target this pathway.

Beyond signaling molecules, the influence of S-glutathionylation on cell structure components is another example of the duality of S-glutathionylation effects. While this modification tends to lower cell growth and motility by inhibiting cytoskeleton polymerization as well as surface adhesion molecules, the positive effects observed on metalloprotease proteins favor cell migration within extracellular tissues. S-glutathionylation induced degradation of adhesion molecules might also play an important role in the regulation of leukocyte transmigration and inflammation.

All modifications depicted in this review are integrated into a diagram to reflect the complexity of S-glutathionylation redox switch in the modulation of EC signaling pathways (Figure 3). Altogether, these findings highlight the wide range of proteins targeted for S-glutathionylation and the diversity of EC functions involved. This process is now recognized as a novel signaling “switch” among other more characterized PTM such as phosphorylation. 

However, the difficulty lies in finding a consistent effect of S-glutathionylation and appreciating the complex mechanism of RONS/antioxidant balance during physiology and pathophysiology. The importance of basal redox signaling in normal physiology could provide a better understanding of why antioxidant therapies failed to meet expectations. Greater appreciation of redox signaling in physiology is of upmost importance since antioxidant treatments in clinical trials have caused unexpected adverse effects. Studies on S-glutathionylation are still at their relatively early stage compared to other PTMs. Another aspect which requires further investigation to appreciate the implications in cardiovascular pathophysiology is how S-glutathionylation interrelates with other oxPTM. A better understanding of the S-glutathionylation process in EC could illuminate the complicated relations linking oxidative stress/redox signaling with CVDs and may also be relevant for other pathologies such as cancer and inflammatory diseases.

## Figures and Tables

**Table 1:** Summary of S-glutathionylation activation (green) or inhibition (red) effects on major molecular players in endothelial cell (EC) function and their physiological consequences.

Protein Name	Protein Type	Glutathionylated Cysteine(s)	Direct Effect(s) on Protein	Physiological Effect(s) in ECs	Reference(s)
4.1. Epigenetics regulators					
Histone H3	Nucleosomal	C110	Not confirmed	Regulation of gene expression via modulating chromatin structure	[13,14]
Sirtuin1	Histone deacetylase	C67 C268 C623	Inhibition of enzymatic activity	Apoptosis - Senescence	[15,16,17]
4.2. Transcription factors					
p65	Transcription factor	Unknown	Inhibition of nuclear translocation	Angiogenesis & cell survival	[18]
p50		C62	Inhibition of DNA-binding activity		[19]
c-jun		C269		Unknown	[20,21]
p53		C124 C141 C182	Inhibition of DNA-binding and protein dimerization	Angiogenesis & cell survival (supposed)	[22]
HIF-1a		C520	Protein stabilization	Angiogenesis & ischemic revascularisation	[23,24]
STAT3		C328 C542	Inhibition of phosphorylation and activity	Anti-angiogenesis and reduced inflammation	[25,26,27,28]
STAT1		C324 C492	Protein activation	Unknown	[28]
Keap1	Nrf2 inhibitor	C434	Inhibition of Nrf2 binding	Antioxidant and anti-inflammatory response via Nrf2 signalling	[29,30]
IKKb	Kinase	C179	Inhibition of kinase activity	Angiogenesis & neovascularisation	[31]
5. Kinases & phosphatases					
LMW PTP	Phosphatase	Unknown	Inhibition of activity	Cell migration and angiogenesis	[32]
PTP1B		C215		Not confirmed	[33,34]
Rac1	Small Rho GTPase	C81 C157		Altered actin structure and barrier function	[35,36]
Rac2		C157	Increased GTP-binding activity	Unknown	[36,37]
Ras	GTPase	C118	Not confirmed	Unknown	[36,38,39]
PKA	Kinase	C199	Inhibition of activity	Alteration of barrier function and blood pressure regulation (supposed)	[40,41]
PKB		Unknown			[40,42,43]
MEKK1		C1238			[40,44]
PKC		Unknown			[40,45]
6. RONS production					
p47 phox	NADPH oxidase	C98 C111 C196	Enhanced protein function	Sustained superoxide production Endothelial dysfunction (supposed)	[46]
Complex I	NADH-ubiquinone oxidoreductase	Unknown			[47]
eNOS	Oxide synthase	C689 C908	Protein uncoupling	Sustained superoxide production Impaired vasodilation and endothelial dysfunction	[48,49,50,51,52]
7. Calcium-dependent channels					
IP3R	Ca^2+^ channel	Unknown (C34 C42 C65?)	Protein activation	Increased [Ca^2+^]_i_ Regulation of Ca^2+^ homeostasis	[53,54,55]
PMCA	Ca^2+^ ATPase pump	Unknown	Protein inhibition		[56]
SERCA2b		C674	Protein activation	Increased Ca^2+^ uptake in ER stores Cell migration and angiogenesis	[57,58,59,60,61,62]
STIM1	Ca^2+^ sensor	C56	Protein oligomerization	Increased [Ca]_i_ via Orai1 activation Mitochondrial dysfunction	[63]
8. Apoptosis and autophagy					
Fas	Death receptor	C294	Enhanced activity	Cell death	[64]
Caspase-3	Protease	C45 (p12) C135 (p17)	Inhibition of proteolytic activity	Cell survival	[65,66]
Caspase-8		Unknown			[67]
Beclin-1	Autophagy-related protein	Unknown	Upregulation of protein activity	Not confirmed	[68]
9. Cell structure and dynamics					
ProMMPs	Metalloprotease	PRCGVPD motif on inhibitory domain	Activation	Angiogenesis and vascular permeability	[69]
ICAM-1	Adhesion receptor	Unknown	Protein degradation	Cell junction disassembly	[70]
Actin	Cytoskeletal	C374	Inhibition of polymerization	Inhibition of cell motility	[71,72,73]
Microtubules		Unknown		Cell growth arrest and apoptosis	[74,75]

## Abbreviations

[Ca^2+^]_i_	cytosolic calcium ion concentrations
AngII	angiotensin II
Ca^2+^	calcium ion
CO	carbon oxide
CVD	cardiovascular diseases
Cys	cysteine
EC	endothelial cell
ECM	extracellular matrix
eNOS	endothelial nitric oxide synthase
ER	endoplasmic reticulum
Grx	glutaredoxin
GSH	glutathione
HDAC	histone deacetylase
HIF-1α	hypoxia-inducible factor 1α
ICAM-1	intercellular adhesion molecule 1
LMW-PTP	Low-molecular-weight protein tyrosin phosphatase
MAPK	mitogen-activated protein kinase
MEK	mitogen-activated protein kinase kinase
MMP	matrix metalloproteinases
NAD+	nicotinamide adenine dinucleotide
NFκB	nuclear factor-kappa B
NO	nitric oxide
NOX	NADPH oxidase
Nrf2	nuclear factor erythroid 2-related factor 2
O_2_	superoxide
oxPTM	oxidative post-translational modifications
PKB	protein kinase B
PTM	post-translational modifications
PTP	protein tyrosine phosphatase
Rac1	ras-related C3 botulinum toxin substrate 1
RONS	reactive oxygen and nitrogen species
Sirt-1	sirtuin 1
STAT	signal transducer and activator of transcription
TGF-β	transforming growth factor beta
TIMPs	tissue inhibitors of metalloproteinases
TNF-α	tumor necrosis factor alpha
VCAM-1	vascular cell adhesion protein 1
VEGF	vascular endothelial growth factor
VEGFR1	vascular endothelial growth factor receptor 1
